# External Corrosion Detection of Oil Pipelines Using Fiber Optics

**DOI:** 10.3390/s20030684

**Published:** 2020-01-26

**Authors:** Nader Vahdati, Xueting Wang, Oleg Shiryayev, Paul Rostron, Fook Fah Yap

**Affiliations:** 1 Department of Mechanical Engineering, Khalifa University of Science and Technology, Sas Al Nakhl Campus, Abu Dhabi 999041, UAE; xuwang@pi.ac.ae (X.W.); paul.rostron@ku.ac.ae (P.R.); 2 Department of Mechanical Engineering, University of Alaska, Anchorage, AK 99508, USA; oshiryayev@alaska.edu; 3 Department of Mechanical and Aerospace Engineering, Nanyang Technological University, Singapore 639798, Singapore; mffyap@ntu.edu.sg

**Keywords:** corrosion sensor, oil and gas pipelines, optical fibers, Fiber Bragg Grating (FBG)

## Abstract

Oil flowlines, the first “pipeline” system connected to the wellhead, are pipelines that are 5 to 30.5 cm (two to twelve inches) in diameter, most susceptible to corrosion, and very difficult to inspect. Herein, an external corrosion detection sensor for oil and gas pipelines, consisting of a semicircular plastic strip, a flat dog-bone-shaped sacrificial metal plate made out of the same pipeline material, and an optical fiber with Fiber Bragg Grating (FBG) sensors, is described. In the actual application, multiple FBG optical fibers are attached to an oil and gas pipeline using straps or strips or very large hose clamps, and, every few meters, our proposed corrosion detection sensor will be glued to the FBG sensors. When the plastic parts are attached to the sacrificial metals, the plastic parts will be deformed and stressed; thus, placing the FBG sensors in tension. When corrosion is severe at any given pipeline location, the sacrificial metal at that location will corrode till failure and the tension strain is relieved at that FBG Sensor location, and therefore, a signal is detected at the interrogator. Herein, the external corrosion detection sensor and its design equations are described, and experimental results, verifying our theory, are presented.

## 1. Introduction

Pipelines are the most practical, economical, and safest way of transporting crude or refined oil and gas (O&G) around the world. A study done by the Fraser Institute [1], comparing safety of transporting oil and gas (O&G) by rail versus pipelines, revealed that both ways are safe but pipelines are the safest transportation mode. Even all living creatures except flatworms, nematodes, and cnidarians have circulatory systems and use veins and arteries (pipelines) to transport blood, oxygen, and nutrients to their bodies. However, from time to time, rupture of the pipelines or veins and arteries can occur. In the case of O&G pipelines, as they are transporting flammable and very hazardous materials, any rupture or defect of the pipeline can potentially result in explosions, fires, release of toxic gases, loss of human lives, property damage, and environmental disasters. Many living creatures and humans have a nervous system, which can detect rupture of veins and arteries when it occurs (acting as a health monitoring system) to warn humans of occurrence of such events, but such health monitoring systems are nonexistent in most O&G fields.

The purpose of this research is to develop a health monitoring system (a corrosion detection sensor) using fiber optics to facilitate detection of external corrosion and help prevent leaks in exposed O&G pipelines. In this paper, an external corrosion detection sensor for O&G pipelines, consisting of a semicircular plastic strip, a flat dog-bone-shaped sacrificial metal plate made out of the same pipeline material, and an optical fiber with FBG sensors, is described. The external corrosion detection sensor and its design equations are described, and experimental results, verifying our theory, are also presented.

### 1.1. Literature Review—Corrosion Prevention

Protective coatings (zinc, epoxy, paint, and other polymers) applied to the outside of O&G pipelines, and cathodic protection are two most commonly used methods [2] by the oil industry to control and inhibit external corrosion of buried pipelines. For above the ground pipelines, which is the focus of this paper, cathodic protection is not an option since the electrolyte (water or soil) is not present and protective coating is the only external protection. The most common types of coatings used in the O&G industry are zinc coating, created during manufacturing of the pipelines and epoxy coating, which is a paint-like substance that seals the surface of the pipeline. Pipeline coating prevents the metal pipe from being in direct contact with the environment, thus extending its life.

Despite the use of coatings, due to mechanical and environmental damages to the coating, external corrosion still takes place on above the ground pipelines. As most oil fields lack a corrosion monitoring system, corrosion can occur undetected.

Another indirect method used to protect pipelines from external corrosion has been pipeline insulation, acting like coatings. Pipeline insulation is used to reduce energy loss, maintain temperature in O&G pipelines, control paraffin waxes from precipitation, and prevent pipelines from freezing and cracking, and is designed to be water tight to prevent infiltration of water from the outside environment onto the pipeline surface; thus, protecting the pipelines from external corrosion but due to mechanical and environmental damages to the insulation, water invariably seeps into the insulation, and pipeline corrosion occurs. Corrosion under insulation (CUI) or corrosion under fire-proofing (CUF) is reported by most O&G and petrochemical companies to be one of their worst nightmares. The cost associated with controlling corrosion is astronomical. On 21 June 2016, PHMSA (US Pipeline and Hazardous Materials Safety Administration) issued an advisory bulletin [3] warning the pipeline industry about Corrosion Under Insulation (CUI). Thermal insulation not only has failed to shield O&G pipelines from external corrosion, but has actually exacerbated the corrosion problem.

Corrosion of low carbon steel pipelines cannot be entirely eliminated but can only be controlled; meaning occurrence of corrosion is a certainty. Thus, there is a definite need for corrosion detection, and inspection. As corrosion is a major threat to O&G pipelines, its inhibition and timely detection are the two key parts of pipeline integrity practice.

### 1.2. Literature Review—Corrosion/Leak Detection Sensors

In the past four years, numerous in-depth review papers have been published [4,5,6,7,8,9,10,11] that show inspection techniques commonly used by the O&G industry for external and internal corrosion detection of O&G pipelines.

For above the ground O&G pipelines without an insulation or with insulations removed, visual inspection, ultrasonic thickness measurement method, Pipeline Inspection Gage (PIG) or Inline Inspection (ILI) tool, and Hydrostatic Pressure Testing (HT) are four main pipeline integrity inspection techniques used by most O&G companies to detect external corrosion and assess if a corroded pipeline is safe to be in operation or not. These techniques have their advantages and disadvantages, and each reflects a different, unique aspect of the overall pipeline integrity management.

The first and the simplest method of inspection is, of course, visual inspection, but it is also the most expensive and time-consuming method. It involves a person walking along the pipeline and checking the surface condition of the pipe, looking for dents, pitting corrosion, metal loss, cracks, and other defects. Visual inspections are usually performed with portable visual scanners (laser scanners), which allow for precise, traceable sizing of surface corrosion at the outer diameter of the pipeline. The task of visual inspection of O&G pipelines becomes harder when insulations are involved. Insulation has to be first removed before the visual inspection and later replaced when visual inspection is complete. This is why this method is labeled as expensive and time-consuming. Corrosion under insulation (CUI), as explained earlier, is one of the most difficult corrosion processes to detect and prevent, as the insulation covers the corrosion problem until it is too late.

When corrosion is found on the surface of the pipeline, ultrasonic thickness measurement method can be used to detect the depth of the corrosion and, at the same time, detect if internal corrosion is also occurring at the same location where external corrosion has been found. Ultrasonic thickness measurement method is a very effective tool in determining local wall thickness of a pipe, but this method is limited to small areas and it takes a long time to use this method to inspect a large area of a pipeline. This method is mostly used to see the severity of corrosion defects when visual inspection shows occurrence of external corrosion.

In-line inspection (ILI) tools, or also called smart pipeline inspection gauges (pigs) travel through a pipeline scanning, measuring, and recording wall thickness, and looking for metal loss, dents, corrosion, deformations, cracking, or other defects [12]. Smart pigs use magnetic flux leakage (MFL) [13] or ultrasonic waves [14] to identify potential problems. The resulting data is then analyzed to diagnose issues and schedule maintenance. Most O&G companies use ILI technology every 3 to 5 years, thus, due to this long inspection interval, the ILI method cannot be considered as a health monitoring system, but instead as an inspection method. ILI testing involves production shutdown which is very costly, even when no corrosion is found during the ILI inspection.

Another technique used by the O&G industry to test pipelines for strength and leaks is hydrostatic testing [15]. This technique is particularly used to test newly laid pipelines for leaks. However, the same technique can also be applied to existing pipelines with defects and corrosion damage. The test involves filling a segment of a corroded pipeline with a liquid, usually water, which may be dyed to aid in visual leak detection, and pressurization of the pipeline to the specified test pressure. The test pressure is normally chosen higher than the working pressure to create a factor of safely. After shutting off the supply valve, the pressure tightness can be tested by observing whether there is pressure loss. The location of a leak can be visually identified since the water contains a dye and repairs can be performed if a leak or severe corrosion is found. Hydrostatic testing involves production shutdown which is again very costly.

To inspect insulated O&G pipelines for external corrosion, in addition to ILIs and hydrostatic pressure testing, the following three techniques are also used; Neutron Backscatter, X-rays or Radiography [16], and Pulsed Eddy Current [17].

In the case of the Neutron Backscatter method, the technique is not used to directly detect external corrosion of O&G pipelines but to detect presence of water underneath the thermal insulation. A radioactive source emits high-energy neutrons into the insulation. If there is moisture in the insulation, the hydrogen nuclei attenuate the energy of the neutrons. If presence of water under the thermal insulation is detected using Neutron Backscatter method, then, most likely, external corrosion under insulation (CUI) is occurring.

When thermal insulation is present, X-rays or Radiography method can be used to detect change in pipe wall thickness due to corrosion. Sections of the pipe wall, suspected of having corrosion, can be exposed to Iridium 192 or Cobalt 60 gamma rays, and the radiation transmitted through the pipe is captured using sensitive films. The sensitive film carries the image of the pipe section and the image can be used to calculate the remaining wall thickness of the pipe. This method is effective in detecting CUI, but it is limited to small area coverage. The radiation hazard to radiography personnel who perform the inspection is also of concern.

The Pulsed Eddy Current method is another inspection technique used to detect corrosion under insulation. Eddy currents are generated in the pipeline wall due to magnetic field produced by a coil. The coil-induced magnetic field is created by applying and controlling the electrical current to the coil. The thicker the pipeline wall, the longer it takes for the eddy currents to decay to zero. This property and technique are used to detect remaining wall thickness of pipelines.

All the techniques mentioned above, are inspection techniques. They are not able to provide real-time or on-demand corrosion monitoring for the O&G pipelines. As the above-mentioned inspection techniques are typically used once every few years, aggressive pipeline corrosion can occur in between inspection intervals without the knowledge of pipeline operators.

The demand for developing a corrosion detection sensor for O&G pipelines is ever increasing due to industry regulations and an aging pipeline network. The fact that most O&G flowlines cannot be pigged or are very difficult to pig, also plays a role in the demand for development of corrosion detection sensors that can be permanently deployed in the field. The industry is still in the search of a sensing solution that could be permanently deployed in the field, does not affect oil production, will be safe in volatile environments, cost-effective, require no or little power, and will not require any alteration or intrusion in the pipeline wall. Many of the existing inspection or health monitoring technologies violate the above-mentioned requirements, but our proposed sensor meets all the mentioned requirements as will be seen later.

Several sensors have been proposed for monitoring of pipelines based on optical fibers. Ren et al. [18] proposed to monitor hoop strain in the pressurized pipe, which will change as the pipe wall gets thinner due to degradation from corrosion or erosion. This solution is suitable for determining both external and internal corrosion [19,20], but it also involves removal of protective coatings and is sensitive to pressure fluctuations during pipeline operations. Lawand et al. [21] proposed a corrosivity sensor that can be placed in the vicinity of the exposed pipeline. This solution was based on Radio-Frequency Identification (RFID) technology and required an inspection crew to walk along the pipeline in order to interrogate each sensor.

In this paper, an external corrosion detection sensor, based on fiber optics and strain change, is proposed. It can be placed on the exposed O&G pipelines and interrogated remotely at any time. The size of the sensor is determined using Castigliano’s second theorem and the sensor design equations are verified using the Finite Element Analysis (FEA) method. The sensor prototype was manufactured and tested in an accelerated corrosion test. The OBR 4600 Optical Back-scatter Reflectometer (OBR) was used as the fiber optic interrogator in the experimental apparatus.

The results obtained from the FEA, closed form equations, and the experiment show excellent correlations. Experimental results prove the feasibility of the proposed sensor. This sensor is able to provide corrosivity environment near the O&G pipeline and help prevent leaks by providing early warning for the operators to perform detailed inspection of a specific location on the pipeline.

The sensor is very safe as it involves only light traveling through the optical fiber. The only challenge is that the proposed corrosion sensor is unable to measure the corrosion rate in real-time, but it is able provide an average corrosion rate when the sacrificial metal element in the sensor fails.

## 2. External Corrosion Detection Sensor

**Figure 1:**
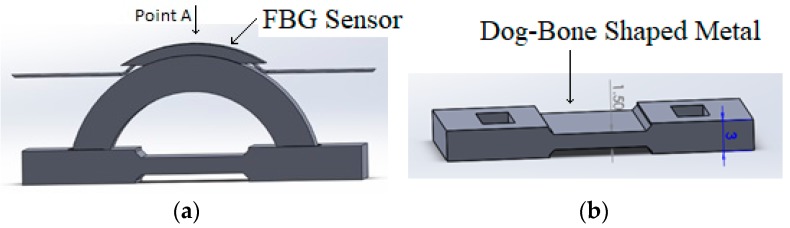
(**a**) External corrosion detection sensor. (**b**) Dog-bone-shaped metal.

The corrosion detection sensor consists of a semicircular shaped plastic curved beam (shown in Figure 1a), attached to a dog-bone-shaped metal component (see Figure 1b), with exact material property as of the pipeline, and an optical fiber with FBG sensors. Each FBG sensor will be glued to a semicircular shaped plastic curved beam at point A, as shown in Figure 1a.

**Figure 2:**
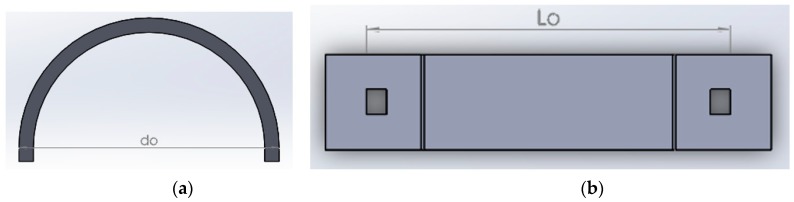
(**a**) Semicircular plastic component. (**b**) Metal similar to pipeline.

As shown in Figure 2, the outer diameter of the semicircular shaped plastic curved beam, d_o_, is chosen to be larger than the length L_o_ by design. When the semicircular shaped plastic curved beam is radially compressed inwards and inserted to the two holes of the dog-bone-shaped metal, the metal and the plastic curved beam at point A are placed in tension.

**Figure 3:**
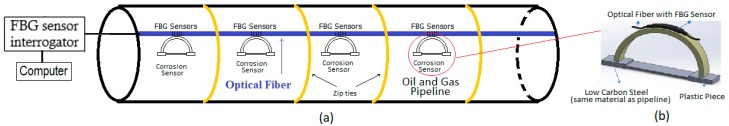
An O&G pipeline with external corrosion detection sensors, spaced every few meters. (**a**) Pipeline with an optical fiber and corrosion detection sensors, (**b**) Corrosion detection sensor.

This tension is also felt by the FBG sensor as it is glued to the semicircular shaped plastic curved beam at point A. Once tension is observed at point A by the interrogator, the interrogator is zeroed. As the dog-bone-shaped metal corrodes, resulting in failure of the dog-bone-shaped metal, the tension at point A is relieved; thus, a signal is picked up by the interrogator. Figure 3 shows how the sensor of Figure 1 will be implemented in the field.

In the field, multiple optical fibers with FBG sensors will be placed on each O&G pipeline. The fiber optic cables with corrosion sensors are attached to the pipeline by simple zip ties, straps, or large hose clamps, see Figure 3. Neither the sacrificial steel material nor the semicircular plastic part shall be rigidly attached to the pipe. Our proposed sensor sits very near the pipeline but not rigidly attached to the pipeline, basically acting as an environmental corrosivity sensor near the pipeline. When the sensor fails due to corrosion, most likely the pipeline may be already corroded since the corrosion sensor is very near to the pipeline. When corrosion is severe at any pipeline location, the dog-bone-shaped metal corrodes at that location and the prestressed semicircular plastic curved beam will break the dog-bone-shaped metal in two pieces, thus a signal is detected by the interrogator at the control room. When a sensor fails at any particular location, the pipeline inspector will visit the pipeline at that location. He or she will conduct a visual inspection first. If corrosion is observed on the pipeline, he or she will use the inspection techniques such as ultrasound technology or eddy current probe and other techniques to further assess the severity of the pipeline corrosion. If repairs are needed, the pipe will be repaired at that location. As the dimension of the dog-bone-shaped metal is known, and, following the ultrasound inspection, the thickness of pipeline corrosion damage is also known, the time to failure is also known, and an average corrosion rate can be calculated. If no corrosion is observed on the pipeline, only the dog-bone-shaped metal is replaced till the next sensor failure.

Note that as the plastic curved beam can be in the deformed state for a long periods of time, seeing extreme ambient temperatures (high and low temperatures) and humidity conditions, the plastic material needs to exhibit no permanent set or creep and should be able to withstand the temperature and humidity condition of the ambient environment. Also, the plastic material needs to have UV resistance, and its mechanical properties should remain constant with aging.

The number of FBG sensors that one can incorporate within a single fiber depends on the wavelength range of operation of each sensor and the total available wavelength range of the interrogator. FBG strain sensors are often given a 4 nm range. Most commercially available interrogators provide a measurement range of 60 to 80 nm. At 80 nm wavelength range, one can only incorporate 20 sensors per fiber. A new interrogator with 160 nm wavelength range and 16 channels has been recently made available to the public. Assuming 4 nm wavelength range of operation for each FBG sensor, and using this new interrogator, at least 40 FBG sensors can be implemented in a single fiber. With a sixteen-channel interrogator, at least 640 FBG sensors can be implemented per pipeline without using any optical switches. Most flowlines are 1 to 4 km long. Assuming a 4 km long oil or gas pipeline (flowline), without using any optical switches, one can monitor corrosion of O&G pipelines every 6.25 m. With the use of optical switches, we can further reduce the distance between the corrosion sensors. If a 3 nm range is used for each FBG sensor, then the total number of sensors that can be used on each pipeline will be 848, meaning every 5 m we can place an external corrosion detection sensor.

Crevice corrosion refers to the localized attack on a metal surface at, or immediately adjacent to, the gap or crevice between two joining surfaces. To eliminate the crevice corrosion between the dog-bone-shaped metal and the plastic curved beam, the ends of the dog-bone-shaped metal can be coated with a thin film of plastic or some anticorrosion coatings. By coating the ends, we force the corrosion to only occur at the center of the dog-bone-shaped metal; thus, eliminating crevice corrosion. Crevice corrosion between the pipeline and the sensor needs to be also avoided. That is why our sensor is not attached to the pipeline but only attached to the optical fibers.

As in most oil fields 3000 to 4000 flowlines maybe present and each pipeline may have anywhere from 600 to 800 sensors, there is a need to keep the cost of the corrosion detection sensor low. To keep the cost of the sensor low, 3D printers can be used to manufacture the plastic semicircular shaped component. The 3D printing technology to print the plastic component is very mature. As for metals, in recent years, major research has taken place with printing metal components and some 3D printing companies claim that they are able to print steel with 0.02% to 2% carbon content. The authors believe in few years’ time, the technology to print all sensor components using 3D printers will be there, and the cost of the corrosion detection sensor will go down as time goes on.

### Sensor Design Equations

To design the corrosion detection sensor of Figure 1 at different sizes, developing a closed form design equation for the above proposed sensor is required. Most optical fibers can only handle strains up to a limit and design equations will thus be necessary to make sure the strain of the optical fiber at the FBG sensor locations does not exceed the manufacturer specified strain limit. To verify the developed closed form design equations, ANSYS finite element software will be used to compare the analytical results with ANSYS results.

**Figure 4:**
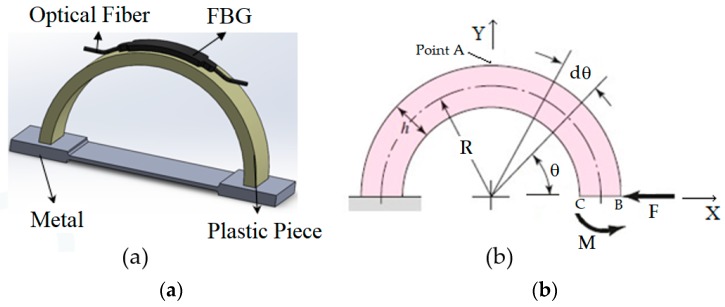
(**a**) Assembled sensor. (**b**) Boundary conditions.

In Figure 2, as we mentioned earlier, the distance d_o_ is larger than L_o_, meaning to insert the semicircular plastic curved beam to the holes of the dog-bone-shaped metal (see Figure 2b), one needs to compress the semicircular plastic curved beam radially inwards. When the semicircular plastic curved beam is compressed radially inwards and then inserted to the dog bone shaped metal piece, we get the picture of Figure 4a. The boundary conditions applied to the plastic curved beam will be as follows, see Figure 4b.

In actual practice, to insert the semicircular plastic curved beam to the dog-bone-shaped metal, a radial displacement in the negative X-direction is given to point B, but to apply that displacement, a force F is required to be applied to move point B in the negative x-direction. The force F causes not only to move point B and surface BC in the negative X-direction but also in the negative Y-direction.

Force F rotates surface BC in the clock-wise direction. A counterclockwise moment M is needed to be applied to the right end of the curved beam in order to bring the slope of surface BC to zero and keep the motion of the BC-surface in the Y-direction to zero.

**Figure 5:**
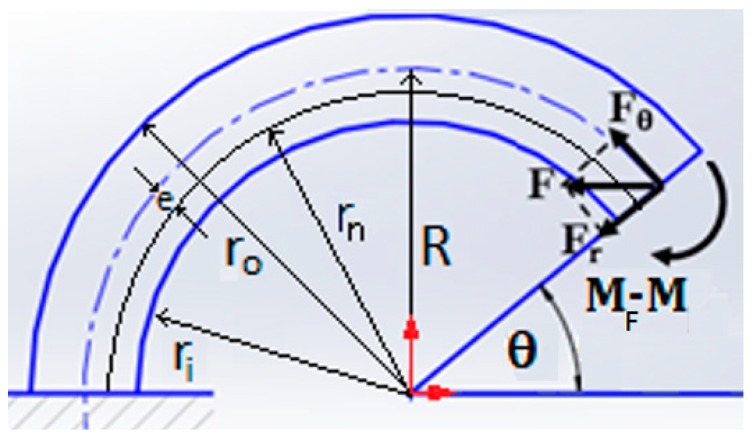
Plastic semicircular curved beam internal forces and moments.

At any angle θ, the internal forces and moments will be as follows, see Figure 5.

The internal forces and moments (shown in Figure 5), at any angle θ, are equal to

(1) $$F_{\theta }=F\sin\theta$$

(2) $$F_{r}=F\cos\theta$$

(3) $$M_{F}=\mathrm{FR}\sin\theta$$

The curved beam stress, at any angle θ, and at any radius r is equal to [22]

(4) $$\sigma =\frac{M_{t}\left(r-r_{n}\right)}{\mathrm{Aer}}-\frac{F_{\theta }}{A}$$

where M_t_ = (M_F_ − M). The strain, at any angle θ, and at any radius r will be equal to

(5) $$\varepsilon =\frac{M_{t}\left(r-r_{n}\right)}{\mathrm{AeEr}}-\frac{F_{\theta }}{\mathrm{AE}}$$

The strain limit of the FBG sensor determines the maximum deflection that can be given to point B. The Castigliano’s second theorem is used to develop the closed form equations.

In Figure 5, for a curved beam with a rectangular cross section, the width is assumed to be W, and the outer and the inner radii are r_o_ and r_i_, respectively. The location of neutral axis is given by Equation (6).

(6) $$r_{n}=\frac{A}{\int_{A}^{\;}\frac{\mathrm{dA}}{r}}=\frac{W\left(r_{o}-r_{i}\right)}{\int_{r}^{\;}\frac{W\left(\mathrm{dr}\right)}{r}}=\frac{W\left(r_{o}-r_{i}\right)}{W\int_{r_{i}}^{r_{o}}\frac{1}{r}\mathrm{dr}}=\frac{\left(r_{o}-r_{i}\right)}{{\mathrm{lnr}|}_{r_{i}}^{r_{o}}}=\frac{\left(r_{o}-r_{i}\right)}{{\mathrm{lnr}}_{o}-{\mathrm{lnr}}_{i}}$$

If the curved beam is sectioned at an angle, θ, see Figure 5, as explained earlier, there will be two forces, F_r_, and F_θ_ and a moment, M_t_, at that section. The total strain energy of the semicircular curved beam from 0 < θ < π, can be calculated by adding four terms, shown below in Equation (7).

(7) $$U=\int^{\;}\frac{M_{t}{}^{2}}{2\mathrm{EAe}}d\theta \;+\;\int^{\;}\frac{F_{\theta }^{2}r_{c}}{2\mathrm{EA}}d\theta \;+\;\int^{\;}\frac{M_{t}F_{\theta }}{\mathrm{EA}}d\theta \;+\;\int^{\;}\frac{{\mathrm{CF}}_{r}^{2}r_{c}}{2\mathrm{AG}}d\theta$$

The first strain energy term in Equation (7) is generated by the moment M_t_, the second term is due to axial force F_θ_, the third term accounts for coupling energy due to M_t_ and axial force F_θ_, and the fourth term is due to transverse shear energy due to radial force F_r_ [23]. The parameter C in the fourth term is the strain-energy correction factor for transverse shear, equal to 1.2 when the cross section is rectangular [24].

Using Equation (7) and conducting a lengthy mathematics, please see reference [22] for more details, we arrive to the following two important equations:

(8) $$u_{x}=u_{\mathrm{xF}}\;-{\;u}_{\mathrm{xM}}=F\left(\frac{{\pi r}_{c}^{2}}{2\mathrm{EAe}}\;-\;\frac{{\pi r}_{c}}{2\mathrm{EA}}\;+\;\frac{{\pi \mathrm{Cr}}_{c}}{2\mathrm{AG}}\right)\;-\;M\left(\frac{2r_{c}}{\mathrm{EAe}}\;+\;\frac{2}{\mathrm{EA}\;}\right)$$

(9) $$u_{y}=u_{\mathrm{yF}}\;-{\;u}_{\mathrm{yM}}=F\left(\frac{2r_{c}^{2}}{\mathrm{EAe}}\;-\;\frac{2r_{c}}{\mathrm{EA}}\right)\;-\;M\frac{{\pi r}_{c}}{\mathrm{EAe}}$$

For the design of Figure 4, u_y_ = 0, and u_x_ is known. Knowing u_x_ and u_y_, force F and moment M can be calculated from Equations (8) and (9). Knowing F and M, we can now calculate the strain ε using Equation (5). The maximum strain occurs at θ = 90 degrees and r = r_o_.

## 3. Equation Validation Using Finite Elements

To validate Equations (5), (8), and (9), ANSYS finite element (FE) software was used to model a semicircular plastic beam, made from PVC material, with following dimensions (see Table 1).

**Figure 6:**
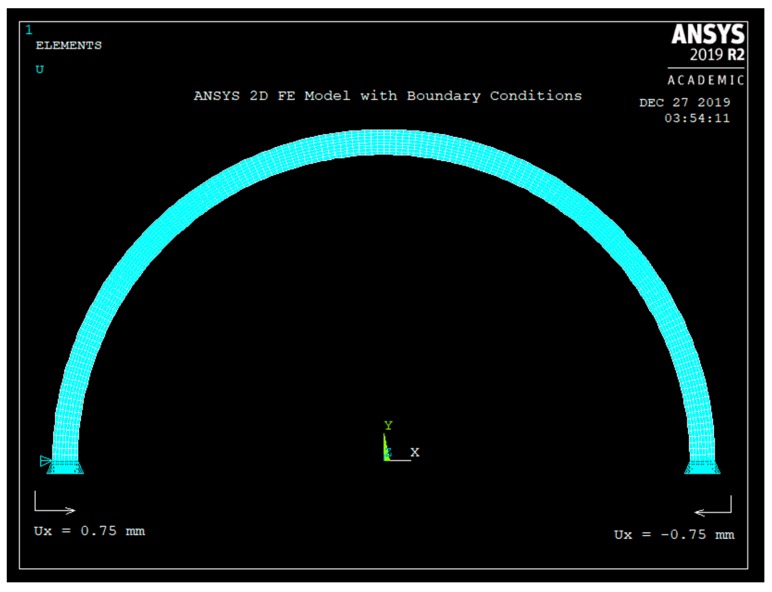
ANSYS Finite Element (FE) Model.

As was explained earlier in Section 2, the semicircular shaped plastic curved beam is chosen to be larger than the dog-bone-shaped metal. To insert the plastic curved beam to the metal, the beam is radially compressed inwards and inserted to the two holes of the dog-bone-shaped metal. The radial inward motion here is 1.5 mm, as shown in Table 1. The ANSYS 2D FE model is shown in Figure 6. Plane183, 2D 8-node element with “plane stress with thickness” option was used to mesh the curved beam. The thickness of the curved beam (thickness is in to the paper) is set as W = 10 mm. Both ends of the curved beam are held fixed in the “u_y_” direction. The left end is given u_x_ = +0.75 mm and the right end is given u_x_ = −0.75 mm motion, simulating 1.5 mm inward radial motion. The FE model of Figure 6 shows eight elements through the radial thickness.

**Figure 7:**
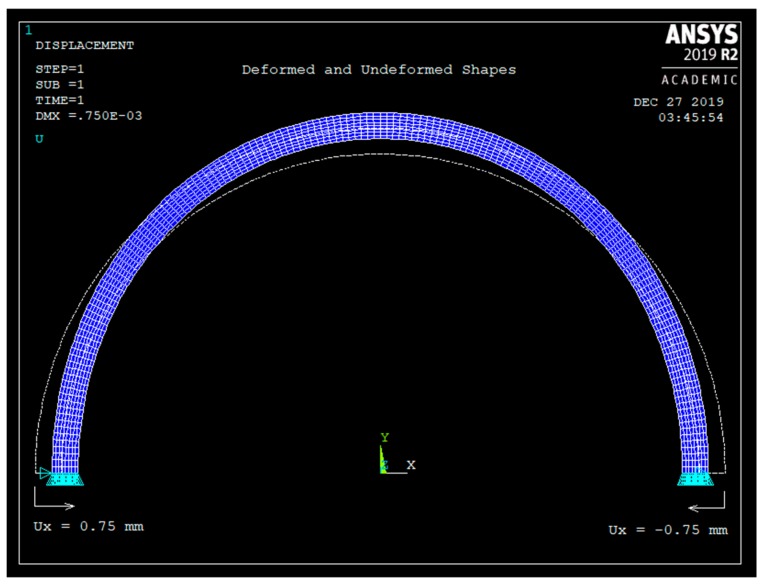
The deformed and the undeformed shapes with boundary conditions.

Figure 7 shows the deformed and the undeformed shape of the semicircular curved beam due to 1.5 mm radial displacement.

**Figure 8:**
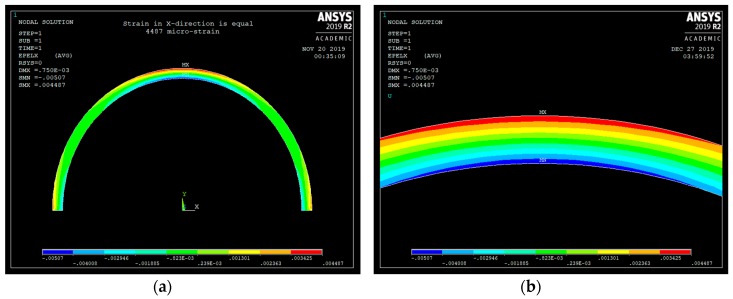
(**a**) Curved beam strain in the x-direction, (**b**) Strain in the x-direction zoomed to point A.

As can be seen from Figure 8, the maximum strain occurs at r = r_o_, at θ = 90 degrees, and at point A (see Figure 4), and it is equal to 4487 με. The maximum strain was calculated using Equations (8), (9) and finally (5) and was found to be 4492 με. The error is only 0.1% (see Table 1). ANSYS FE stress analysis validates the derived equations. Equations (1)–(9) can now be used to design the corrosion sensor of Figure 4.

## 4. Experimental Validation

**Figure 9:**
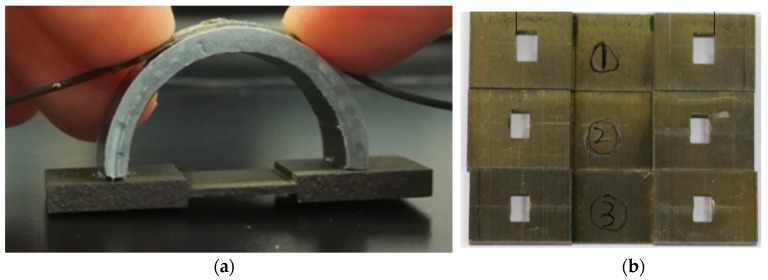
(**a**) Actual corrosion detection sensor. (**b**) The dog-bone-shaped metals.

**Figure 10:**
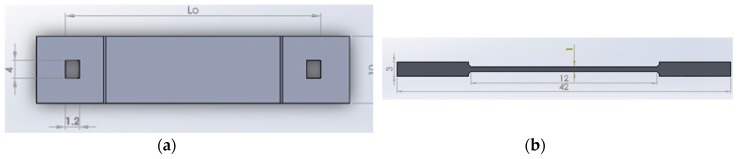
Dimensions of the dog-bone-shaped metal (Lo = 29.24 mm). (**a**) Top view; (**b**) Side view.

**Figure 11:**
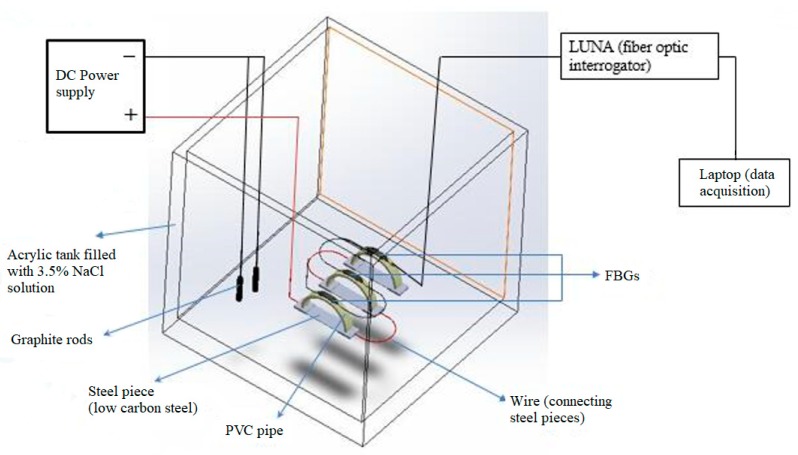
Schematic of the experimental test setup to test the proposed corrosion detection sensor.

**Figure 12:**
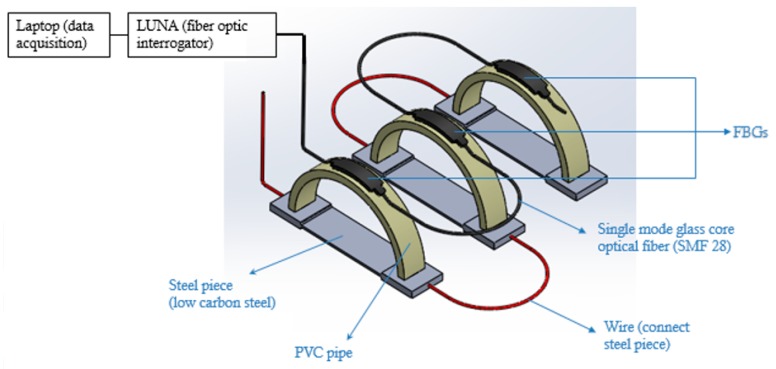
Schematic of details of proposed sensor.

Figure 9 shows the actual corrosion detection sensor and the dog-bone-shaped metals (low carbon steel), made from the same O&G pipeline material. The semicircular curved beam is made of PVC for the experiment, but long-term, it will be constructed using a 3D printer. Three corrosion detection sensors were constructed using the three dog-bone-shaped metals shown in Figure 9. The dimensions of the dog-bone-shaped metals are shown in Figure 10. The three corrosion sensors were placed in series as shown in Figure 11; Figure 12. Figure 11; Figure 12 show the experimental apparatus used for validating the performance of the proposed external corrosion detection sensor.

The optical fiber used in our experiment is a single mode fiber (SMF 28) with 5 FBG sensors, with FBG sensors 1 m apart. The first FBG Sensor is located at 0.5 m from the end of the optical fiber that connects to the interrogator. Glue was used to attach the outer surface of the PVC rings to the bottom surface of the FBG sensors (see Figure 9; Figure 12). To make sure the bond between the FBG sensor and the semicircular plastic curved beam remains intact at high temperature of the pipeline (≤150 °C) and high humidity of the desert for prolonged periods of time, the authors of [25] recommend a compound, based on a combination of ceramic fillers with an epoxy binder that is applied with a brush technique, and this compound can withstand temperatures in the region of 260 °C and humidity of 75%.

The optical fiber was connected to an interrogator with wavelength range of 1270−1340 nm. The connection termination type between the optical fiber and the interrogator was FC-APC.

**Figure 13:**
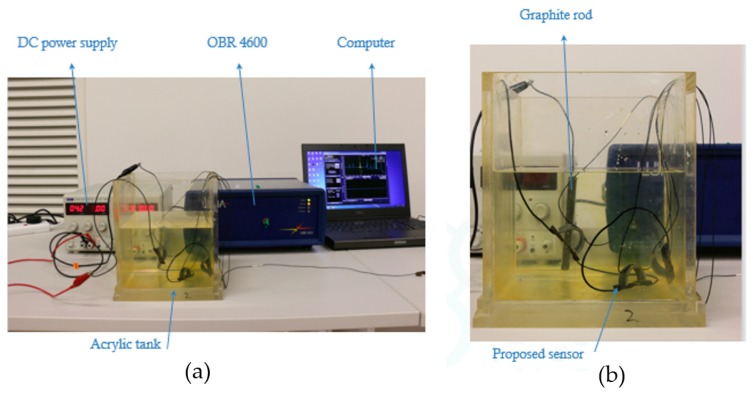
(**a**) Experimental test set-up. (**b**) and details of the apparatus inside acrylic tank.

Most O&G pipelines are made of API 5L X42 to X70 material with carbon content from 0.16% to 0.28% (mild or low carbon steels). Other mild or low carbon steel materials are also used in the O&G industry. The dog-bone-shaped metal pieces are required to be made from the same pipeline material. For our experiment, API 5L X65 material was used. As can be seen from Figure 11, the dog-bone-shaped metals are linked to one another using wires. Wires are soldered to the dog-bone-shaped metals and the solder joints were coated with an anticorrosion coating. Figure 13 shows the entire experimental test set-up consisting of a laptop, an interrogator, corrosion cell, and a power supply.

**Figure 14:**
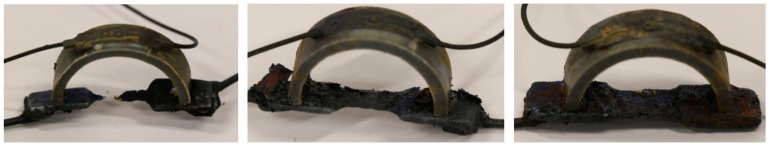
The three corrosion sensors after accelerated corrosion failure.

A DC power supply (4 V and 1 A) was used to accelerate the corrosion reaction. Graphite rods were the cathode and the low carbon steel pieces were the anode, all of them are placed into 3.5% by weight NaCl solution which is regarded as the electrolyte. The thinnest section of the dog-bone-shaped metals is in the middle, with thickness of 1 mm. It was at this location where the corrosion failure first occurred. The experiment kept running until complete failure of one of the sensors. It took almost 14 h to corrode one of the sensors. The three corroded sensors are shown in Figure 14.

**Figure 15:**
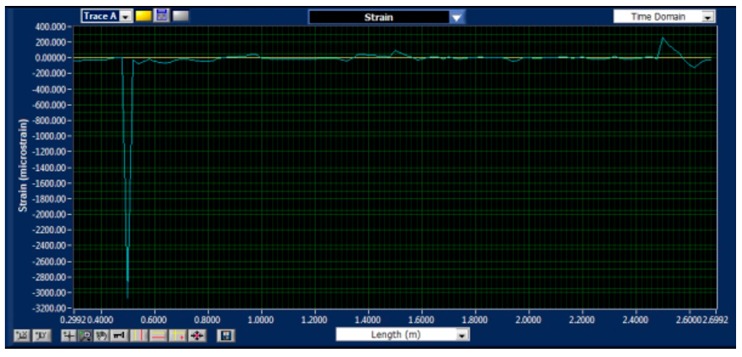
(**a**) Yellow trace displays strain before sensor failure. (**b**) Blue trace displays strain after sensor failure due to corrosion.

As was explained on page 10, the optical fiber used in our experiment is a single mode fiber (SMF 28) with five FBG sensors, with FBG sensors 1 m apart. The first FBG Sensor is located at 0.5 m from the end of the optical fiber that connects to the interrogator. Thus, the first FBG Sensor is located at 0.5 m, the 2nd FBG sensor at 1.5 m, the third at 2.5 m, and so on. In our experiment, only three FBG sensors out of five were used. Figure 15 shows the strain observed by all the FBG sensors before and after the failure of the first corrosion sensor. The yellow color trace is the strain observed by all the FBG sensors before the failure of the 1st corrosion sensor, which is about zero and the blue color trace is the strain observed by all the FBG sensors after the failure of the 1st corrosion sensor. The y-axis shows the strain (in micro strain) of all the FBG sensors and the x-axis indicates the location (in meters) of the FBG sensors. In the upper right corner of the Figure 15, the graph indicates “time domain”. This implies that the strain at each FBG sensor location can change with time. If we would have continued the accelerated corrosion, we would have had the failure of the 2nd or 3rd corrosion sensors and in Figure 15, we would have seen additional peaks, as time goes by.

The peak value of the strain, at 0.5 m, where the first FBG sensor is located, is −3100 με. When the semicircular plastic curved beam is inserted to the dog-bone-shaped metal, point A (where the FBG sensor is attached to) will be strained. The strain at point A will be in tension. We tared (zeroed out) that strain in the interrogator. However, when the dog-bone-shaped metal corrodes, the semicircular plastic curved beam is released thus negative strain is observed on the interrogator display monitor.

It was not necessary to tare the strain. We could have done the opposite, meaning not zero out the strain and leave the strain at point A as it is and when the dog-bone-shaped metal is corroded, the strain at point A would have gone back to zero.

## 5. Discussion

The external corrosion rate of O&G pipelines, based on NACE Standard (NACE RP0-502: Pipeline External Corrosion Direct Assessment Methodology), is ~0.4 mm/year [26]. Due to this low corrosion rate, larger diameter O&G pipelines are inspected every 3 to 5 years. Pipeline pigging and data analysis is very costly, and due to this high cost, most O&G companies have opted for 5-year inspection interval rather than the shorter ones. The smaller diameter pipelines (flowlines) are not even pigged due to diameter of the pipeline being small and no launch and retrieval stations being available to launch and retrieve the PIG. Even if the flowlines were piggable, since pigging occurs every 5 years, if there is an aggressive corrosion occurring due to the change in the pipeline environment or surrounding, O&G industry is vulnerable to possible field accidents in between inspection interval.

As, in normal conditions, the external corrosion rate of O&G pipelines is low, real-time corrosion monitoring is not really necessary, but as long as corrosion state of the pipelines are monitored monthly or every few months, this monitoring protects the O&G industry against aggressive pipeline corrosion. Our proposed sensor can provide corrosion state of the O&G pipelines, as and when needed. Our sensor is basically monitoring the corrosivity environment near the O&G pipelines.

In this paper, we embarked upon designing a very low-cost corrosion detection sensor for O&G pipelines, requiring no power. The sensors are passive, very simple, and they are permanently deployed in the field, unlike ILIs, ultrasound probes, X-ray, or radiography tools. The sensor involves a semicircular plastic component, a sacrificial dog-bone-shaped metal made from the same pipeline material, and optical fibers. The optical fibers have up to 20 to 40 FBG sensors per optical fiber, depending on which type of interrogator used. The optical fibers are attached to the gas and oil pipelines using zip ties, straps, or large hose clamps and our sensors are attached to the FBG sensors. When corrosion is severe at any pipeline location, the dog-bone-shaped metal corrodes at that location and eventually fails and thus a signal is detected by the interrogator at the control room. Once a signal is picked up at the control room, inspection personnel will visit the pipeline at that location and conduct visual inspection first, and possibly ultrasound, X-ray or radiography inspection. If the corrosion is not severe, the dog-bone-shaped metal is replaced until the next failure. If severe corrosion is observed at any pipeline location, the pipeline is inspected using ultrasound probes, or X-rays, or eddy current probes or other inspection methods. Depending on the severity of the corrosion, pipeline may be repaired and the metal dog-bone-shaped metal is replaced till the next sensor failure. In the proposed corrosion monitoring system of this paper, all the communication between the corrosion detection sensors and control room is through the optical fibers. As only light is involved, and there are no batteries or electricity of any kind (since interrogator sits inside the control room), this corrosion monitoring system is very safe.

The thickness of the sacrificial dog-bone-shaped metal was chosen to be 1 mm. With corrosion rate of 0.4 mm/yr, it would take 2.5 years for the sacrificial dog-bone-shaped metal to corrode completely through the 1 mm thickness. Two and a half years is the mid-span of the 5-year inspection interval. If after 2.5 years, no signals are observed on the remote interrogator for any of the corrosion sensors, then one can conclude the corrosion rate is less than 0.4 mm per year for that pipeline. When few sensors fail earlier than 2.5 years, then it implies we are having aggressive corrosion occurring at some specific pipeline locations. Our proposed sensor is very helpful to the pipeline operators as they can now go to those specific locations and find out why there is a higher rate of corrosion at those locations. Steps can be taken at those high corrosion rate locations to lower the corrosion rate back to 0.4 mm/yr or lower.

If there is a leakage of the crude oil, as crude oil is hot, the heat can create tension strain on the FBG sensors thus a signal can be picked up by the interrogator in the control room. The proposed sensor of this paper can not only detect occurrence of pipeline corrosion but also the pipeline fluid leakage.

Ground settlement is a geological phenomenon of ground elevation changing (vertical movement of the ground) caused by the compression of earth’s crust surface soil due to natural and unnatural events. Caving in or sinking of the ground is one form of ground settlement. If the ground caves in, naturally, the O&G pipelines foundations will also cave in. Our fiber optic-based corrosion monitoring system proposed in this paper, most likely, does not capture ground settlement since neither the optical fiber nor the corrosion sensors are rigidly attached to the pipeline unless the ground cave-in is deep. There are few O&G companies already using optical fibers on their O&G pipelines to monitor the potential security risks, detect pipeline temperature, and detect ground settlement. For those companies, presently having optical fibers on their pipelines, it makes sense to bundle our corrosion detection system to their existing fiber optics monitoring system to also detect pipeline corrosion.

## 6. Conclusions

The grating length of most FBG sensors is anywhere from 5 to 20 mm. If the grating length is too large, the strain experienced by the FBG sensor won’t be the strain of point A but the average strain near point A (See Figure 4b). The FBG sensor, used in our experiment, had a grating length of 10 mm. The FBG sensor was picking up the average strain underneath the FBG sensor and not strain at point A only, and that is why the experimental tension strains are below the predicted ANSYS strain results.

Due to the differences in physical–mechanical properties of the matrix material and the FBG sensor, and the adhesive, the strains measured by the FBG sensor may not be equal to the actual strains experienced by the matrix material. When the FBG sensor is glued on to the PVC semicircular curved beam, the area of moment of inertia is changed and the FBG sensor thickness, material and adhesive used can have some impact on the actual measured strain.

The exact value of strain obtained at point A is not important as long as the strain is below the strain limit of the optical fiber and the strain is large enough to be detected by the interrogator; thus, the proposed sensor of this paper can be used to detect occurrence of pipeline corrosion and pipeline leakage, and provide average pipeline corrosion rate.

## Figures and Tables

**Table 1:** Mechanical property and model geometry.

Parameters	Values, SI Units
Inner radius, r_i_	14.22 mm
Outer radius, r_o_	15.37 mm
Thickness, t	1.15 mm
Width, W	10 mm
Young’s Modulus of PVC, E	3.4 GPa
Poisson’s ratio, ν	0.4
End deflection given to the right end, δ	1.5 mm
Strain, ε_x_, obtained from ANSYS, µε	4487
Strain, ε_x_, calculated from Equation (5), µε	4492
Error	0.1%
